# Nutraceutical Content and Biological Properties of Lipophilic and Hydrophilic Fractions of the Phytocomplex from *Pistacia atlantica* Desf. Buds, Roots, and Fruits

**DOI:** 10.3390/plants13050611

**Published:** 2024-02-23

**Authors:** Nabila Belyagoubi-Benhammou, Larbi Belyagoubi, Assia Benmahieddine, Asma El Zerey-Belaskri, Gabriele Di Marco, Alessia D’Agostino, Antonella Canini, Angelo Gismondi

**Affiliations:** 1 Natural Products Laboratory, Department of Biology, Faculty of Natural and Life Sciences, Earth and Universe, University Abou-Bekr Belkaïd, Tlemcen 13000, Algeria; belyagoubi_larbi@yahoo.fr (L.B.); physiopathologiste149@gmail.com (A.B.); 2 Laboratoire de Biotechnologie des Rizobia et Amélioration des Plantes, Faculté des Sciences de la Nature et de la Vie, Université Oran1 Ahmed Ben Bella, Oran 31000, Algeria; asma.elzerey.belaskri@gmail.com; 3 Department of Biology, University of Rome Tor Vergata, Via della Ricerca Scientifica 1, 00133 Rome, Italy; gabriele.di.marco@uniroma2.it (G.D.M.); alessia1211@hotmail.it (A.D.); canini@uniroma2.it (A.C.)

**Keywords:** Atlas pistachio, aqueous extract, plant oil, antioxidant potential, antimicrobial activity, HPLC-DAD, GC/MS

## Abstract

The aim of the present investigation was to obtain 12 aqueous extracts and 1 oil from *Pistacia atlantica* Desf. subsp. *atlantica* specimens. The samples differed for processed plant organs (i.e., roots, buds, and fruits), gender and geographical station of the collected trees. Total phenols, flavonoids, and condensed tannins were determined, revealing that bud extracts exhibited the highest phenolic content (386.785 ± 16.227 mg GAE/g DM), followed by fruit and root preparations. Similar results were detected for flavonoids and tannins, whose quantitation ranged from 0.014 ± 0.005 to 74.780 ± 9.724 mg CE/g DM and from 0.037 ± 0.003 to 14.793 ± 0.821 mg CE/g DM, respectively. The biochemical profile of the extracts was further characterized by HPLC-DAD, in terms of specific phenolics. This analysis identified gallic acid as a typical metabolite for ripe fruit, while hydroxytyrosol for female roots and male buds. In parallel, *P. atlantica* fruit oil was profiled by GC-MS analysis, which detected 37 lipophilic components, including palmitic acid (the major component, ~55%), anacardol, tetradecanol, arachidic acid, squalene, and some terpenes. The samples revealed interesting antioxidant activity, with EC_50_ values ranging from 0.073 ± 0.001 to 193.594 ± 28.942 mg/mL and from 0.029 ± 0.001 to 103.086 ± 20.540 mg/mL, in that order, for DPPH and reducing power assays. Concerning the total antioxidant capacity, the results ranged from 0.053 ± 0.008 to 51.648 ± 1.659 mg AAE/g DM. Finally, the antimicrobial potential of the plant extracts was estimated against 7 bacterial species and 2 fungal strains, known to be human pathogens, demonstrating a good antibiotic effect for the bud extracts. All these findings strongly suggest that *P. atlantica* would represent a natural reservoir for novel additives to be used in therapeutic, food, and cosmetic products.

## 1. Introduction

The widely diversified flora and abundant plant biodiversity in Algeria are well-known. Indeed, ethnobotanical and folk medicinal practices based on the endemic species of this nation have been exercised for a very long time. Indigenous tribes have long traditions in this regard, processing about 4000 taxa, 917 genera, and 131 families [1].

*Pistacia atlantica* Desf., also called “Atlas pistachio” or “Mount Atlas mastic tree”, is one of the most diffused species and typical of Algeria, as well as other countries from North Africa and the Middle East [2]. This tree is suitable for arid and semi-arid areas, especially uplands [3]. *P. atlantica* has a wide range of uses, in both food and pharmaceutical fields; for example, its leaves, fruits, and other aerial parts are used to cure abdominal pain, discomfort, dyspepsia, and peptic ulcer [4]. The seeds are the edible parts that have historically been employed as food. They can be eaten raw, roasted, or as an ingredient in a great variety of dishes and desserts. However, another notable product derived from the aerial parts of this tree is the resin, also known as mastic. This substance has been used in culinary applications, such as a flavoring agent, and as a chewing gum component [5]. Seeds and resin have been also utilized in medicinal preparations against gastrointestinal, liver, splenic, neurological, cardiac, psychological, respiratory, urogenital, and dermatologic disorders [2,6,7]. Due to their content of bioactive compounds with potential health benefits, *P. atlantica* leaves may be also used in phytotherapy, beyond classic recipes, like salads and bread [5,8,9].

Numerous studies have been conducted to demonstrate the wide range of pharmacological activities at the expense of *P. atlantica*, including antibacterial [10,11,12], antioxidant [11,12,13,14], antidiabetic [14,15,16], anti-inflammatory [12,17], hepatoprotective [18], antitumor [19,20], anti-acetylcholinesterase [21,22], and antihyperlipidemic effects [23]. In fact, there are various chemical compounds in this plant species that may justify its potential therapeutic role, including triterpenoids, flavonoids, other phenolic compounds, tannins, fatty acids, and phytosterols [4,24]. Obviously, these compounds and their concentrations can change based on the basis of the environment of growth for the plant and the extraction methods used to isolate them.

In order to demonstrate the exploitation of aqueous extracts from various parts of *P. atlantica* used by the Algerian population, this study, which is an extension of previous research [11,12], demonstrated the phytochemical profile and the potential biological activity of aqueous extracts from this plant, in function of the plant tissue (i.e., root, bud, and fruit), harvest area, and plant gender. Furthermore, an analysis was conducted on the chemical composition of the oil extracted from ripe Atlas pistachio fruits as well as its antibacterial and antioxidant characteristics.

## 2. Results

### 2.1. Extraction Yield

To the best of our knowledge, this report is considered the second study to assess the effect of sex and harvest site on the phytochemicals produced in buds, roots, and fruits of *P. atlantica* from Algeria, beyond Benmahieddine et al. [11]. As shown in Table 1, the yields of bud aqueous extracts, which vary between 27.162 ± 0.859% and 35.005 ± 4.631%, were the highest compared to those from other parts of the plants. In terms of harvest site and gender, the results were not statistically significant. Several studies have reported the effect of the extraction solvent on the content of phenolic compounds [25], indicating that less polar solvents are usually prone to determine high extraction yields, although a great contribution depends on the nature of the molecules of interest. For the unripe fruits, the samples harvested at Safsaf (14.892 ± 1.700%) showed a yield two-fold higher than that from the Oudjlida samples (6.88 ± 0.332%). About the plant oil isolated from Oudjlida ripe fruits, the yield was recorded as equal to 27.603 ± 1.766%. This content remains higher compared to those by male and female leaves reported in Gourine et al. [26], where the component of fatty acids was in a range of 0.8–5.1%. On the other hand, our results were similar to those reported for *P. atlantica* by Guenane et al. [27,28] but weaker than those found by Yousfi [29] and Matthäus and Ozcan [30]. It should be noted that the oil yield increases significantly during fruit maturation, which could be due to the accumulation of fatty acids during the ripening [31,32,33].

As shown in Table 1, the phenolics extracted from *P. atlantica* oil represented a very small percentage of this product (0.12 ± 0.014%), in agreement with Bentireche et al. [34], who reported that this value decreases during fruit ripening from 0.574% to 0.062%. According to the literature, the present contribution would be the third work describing the extraction of phenolic compounds from *Pistacia* fruit oil, after Bentireche et al. [34] and Belyagoubi-Benhammou et al. [35] who worked on the same species and on *P. lentiscus*, respectively.

### 2.2. Determination of Total Phenolics, Flavonoids, and Condensed Tannins

The content of total phenolics, flavonoids, and condensed tannins in the several extracts from *P. atlantica* are shown in Table 2. Compared to the root and fruit samples, the highest phenolic contents were observed in the bud extracts, with a peak value recorded in the male trees from Oudjlida (284.133 ± 37.640 mg GAE/g DM) and Ain-Fezza (386.785 ± 16.227 mg GAE/g DM). Indeed, Oudjlida female buds showed a total phenolic content of just 89.254 ± 2.366 mg GAE/g DM. The flavonoid content followed a similar trend in the same samples: 22.685 ± 0.067 CE/g DM for the Oudjlida female buds, 37.677 ± 0.239, and 74.780 ± 9.724 CE/g DM for the male trees from Oudjlida and Ain-Fezza, respectively. Regarding condensed tannins, the highest contents were recorded in bud samples, with a range from 12.806 ± 0.735 to 14.793 ± 0.821 CE/g DM. This richness in phenolic compounds found in the buds finds support in other published scientific evidence [11,12,36,37].

The total phenolic (86.337 ± 1.232 mg GAE/g DM) and flavonoid (14.641 ± 1.428 mg CE/g DM) content of Oudjlida ripe fruits was higher than those measured from unripe fruits and roots. Furthermore, unripe fruits from Oudjilida showed almost comparable levels of total phenolic and flavonoids (56.287 ± 1.442 mg GAE/g DM and 9.535 ± 1.034 mg CE/g DM, in that order) to those measured for the same samples collected at Safsaf site (that is, respectively, 59.897 ± 0.553 mg GAE/g DM and 6.710 ± 0.047 mg CE/g DM). For condensed tannins, these samples revealed low concentrations. About roots, the female plants recorded the highest doses of phenolic compounds, compared to male ones for the Oudjilida and Safsaf sites.

For the plant oil, the concentrations of total phenolic, flavonoid, and condensed tannins were 0.269 ± 0.032 mg GAE/g DM, 0.014 ± 0.005, and 0.037 ± 0.003 mg CE/g DM, respectively. These results are high compared to those of Bentireche et al. [34], who have reported a decrease in phenolic compounds (including flavonoids) with fruit ripening. By contrast, Belyagoubi-Benhammou et al. [35] have revealed an elevated content of total phenolics (0.81 mg GAE per gram of *P. lentiscus* oil) compared to this work.

### 2.3. HPLC Phytochemical Profile

The chemical composition of *P. atlantica* extracts was investigated by HPLC-DAD analysis to identify and quantify specific phenolic metabolites (i.e., phenols and flavonoids) in these samples and link them to potential biological properties. In detail, this analysis was carried out only on the extracts of ripe fruits from Oudjlida station, female roots from Safsaf station, and male buds from Ain-Fezza, as shown in Table 3, because they were the richest samples in phenols and flavonoids of the series according to our previous spectrophotometric assays (see Table 2). Five phenolic compounds were successfully identified in the extract of Oudjlida ripe fruits. They included four phenols (i.e., gallic acid, chlorogenic acid, ellagic acid, and hydroxytyrosol) and one flavonoid (i.e., rutin). Concerning Safsaf female roots extract, only five phenols were detected (i.e., *p*-coumaric acid, caffeic acid, chlorogenic acid, dimethyl-allyl caffeic acid, and hydroxytyrosol). Finally, for the Ain-Fezza male bud extract, nine phenolic compounds were found. They included five phenols (i.e., chlorogenic acid, caffeic acid, *p*-coumaric acid, hydroxytyrosol, and ellagic acid) and four flavonoids (i.e., rutin, quercetin-3-*O*-arabinoside, kaempferol-3-*O*-glucoside and quercetin). Phenolic ester caffeic acid, myricetin, genistein, kaempferol, apigenin, chrysin, and galangin were not detected under these conditions in any extracts.

Among them, hydroxytyrosol represented the dominant compound in both root and bud aqueous extracts. In a previous work, hydroxytyrosol, ellagic acid, rutin, and dimethyl-allyl caffeic acid were the most abundant molecules in hydro-methanolic extracts from *P. atlantica* buds [11]. In the same study, hydroxytyrosol and rutin were dominants in red fruit extract. Compared to this investigation, hydroxytyrosol (12.01 µg/g) and gallic acid (112.94 µg/g) were the most concentrated phytochemicals in aqueous extracts of mature fruits. This variability was attributed to the wide differences in the solvent used for extraction, part of plant, and maturity stage of fruits. It is known that the phytocomplex generally works in synergy rather than per singular components [38].

### 2.4. Chemical Composition of P. atlantica Ripe Fruit Oil

Table 4 shows the lipophilic profile of *P. atlantica* fruit oil. The major part of the compounds detected by GC-MS in our study were identified for the first time in *P. atlantica*. In detail, a total of 33 analytes were revealed; in this extract, the most predominant metabolite was a fatty acid, that is palmitic acid, with a relative abundance value of 55.180%. Moreover, fruit oil contained a considerable amount of anacardol (9.0603%), tetradecanol (9.1952%), arachidic acid (8.2885%), squalene (8.1807%), oleic acid (3.6240%), linolenic acid (1.5955%), stearic acid (1.2052%), and erucic acid (1.1025%). Beyond vitamin E (0.5943%) and tetradecenal (0.4813%), numerous minor components, whose concentrations varied between 0.0002 and 0.2304%, were found. Interestingly, among them, various terpene compounds (e.g., citronellol, limonene, and alpha-pinene) and an azulene derivative were recorded. Our results are similar to those reported by Guenane et al. [27] and Bentireche et al. [34]. These authors documented that the content of palmitic acid in *P. atlantica* fruit increased during ripening, together with the reduction of the ratio between stearic (C18:0) and arachidic (C20:0) acid. Differently from our evidence, at full maturity, *P. lentiscus* fruit oil showed oleic acid as the main fatty acid, followed by palmitic and linoleic acids [35,39]. Moreover, it has been verified that the saturated fatty acids and polyunsaturated fatty acids decreased significantly while monounsaturated fatty acids increased considerably during the maturation of Coriander fruits [31]. However, all this variability could be explained by various factors influencing the plant material and its extract (e.g., environmental biotic and abiotic factors, soil properties, exact ripeness stage, and harvest period). Finally, the low levels of vitamin E were corroborated by the literature, reporting that different classes of tocopherol decreased their content in mature fruits [27,28].

### 2.5. Antioxidant Property of Extracts

Results of the antioxidant activity of extracts, evaluated by three spectrophotometric assays, are shown in Table 5. The highest DPPH scavenging effect was observed in the extract of ripe fruits from the Oudjlida station (EC_50_ = 0.073 ± 0.001 mg/mL), followed by Safsaf female root extract (EC_50_ = 0.106 ± 0.001 mg/mL), and Ain-Fezza male bud extract (EC_50_ = 0.129 ± 0.001 mg/mL). Concerning the total antioxidant capacity (TAC) and reducing power (RP) tests, the Ain-Fezza male bud sample held the highest activity (51.648 ± 1.659 mg AAE/g DM, EC_50_ = 0.029 ± 0.001, respectively). For fruit and root extracts, in these assays, the same trend was observed in DPPH analysis. The EC_50_ values of reducing power ranged from 0.056 ± 0.002 to 2.543 ± 0.228 mg/mL. These results were comparable to those reported by Toul et al. [37] and Benmahieddine et al. [11] on hydro-methanolic preparations from *P. atlantica* organs and concerning DPPH and TAC tests. On the other hand, the same results appeared high compared to those mentioned in the works of Benmahieddine et al. [12] on hydro-methanolic leaf-bud extracts (EC_50_ = 0.136 ± 0.003 mg/mL). Generally, Ain-Fezza male bud extract, Safsaf female root extract, and Oudjlida ripe fruit extract revealed high antioxidant activities, probably due to the high content and variability of phenolics, such as Gallic acid and Hydroxytyrosol, recorded in these samples by our previous spectrophotometric and HPLC-DAD analyses. In fact, these compounds are well-known for their free radical scavenging and metal-chelating properties [40]. In particular, their antioxidant power is mainly due to the ability to scavenge reactive species and neutralize radicals [41].

### 2.6. Antimicrobial Activity

This part of the study was conducted to evaluate the antimicrobial activity of the various aqueous extracts obtained from *P. atlantica* tissues against several human bacterial and fungal pathogens (Table 6). This biological effect varied according to sampling station, plant organ subjected to extraction, and strain sensibility. The bud extracts showed good in vitro antimicrobial activity against Gram-positive and Gram-negative bacteria, except for *K. pneumoniae*. *S. aureus* had the strongest inhibitory effect, with inhibition zones ranging in diameter from 16 to 23 mm. In addition, our results indicated that the bud extracts could potentially be sources of anticandidal agents, showing the highest inhibition zone diameter against this pathogen (i.e., 15.75 mm with a charge of 3 mg/disc of the extract from Ain-Fezza male plants). Our findings were in line with those of Benmahieddine et al. [11,12], who have reported that *P. atlantica* bud extracts possess a strong bioactivity against pathogenic bacteria and a marginally antifungal effect.

The six different root extracts revealed good antimicrobial activity against *S. aureus*. With the exception of the extract from Oudjlida female roots on the two species of *Bacilus*, the other samples showed a surprising absence of antimicrobial activity.

The results demonstrated also that unripe fruit extracts from Oudjlida were active only against *S. aureus* (24 mm) and *M. luteus* (8.5 mm) at a charge of 3 mg/disc.

*P. atlantica* fruit oil exhibited a considerable antibacterial effect against Gram-positive bacteria (i.e., *S. aureus*, *B. cereus,* and *B. subtilis*), while no bioactivity on Gram-negative bacteria and fungi was observed at the expense of this sample. Several works support this evidence [42,43].

The results of MIC and MBC/MFC of the investigated samples are shown in Table 7. It was found that the aqueous extracts exhibited both bactericidal and fungicidal properties at a concentration equal to 75 mg/mL or more, except for the extracts of Ain-Fezza male buds which showed the lowest MBC values against *S. aureus* and *B. subtilis* and *C. albicans* CIP 444 (i.e., 0.293, 0.293 and 1.875 mg/mL, respectively). Oudjlida female bud extracts also inhibited *S. aureus* and *E. coli* at low MBC values; 1.465 and 1.875 mg/mL, respectively. In general, MIC values of the extracts varied greatly between 0.036 mg/mL and 75 mg/mL. Findings regarding the antimicrobial activity of the plant extracts indicate that, of the nine pathogenic strains that were tested, *S. aureus* was the one that showed the greatest sensitivity to fruit extracts from the three origins, Oudjlida (ripe and unripe) and Safsaf (unripe), (18, 24 and 26 mm, respectively) at a charge of 3 mg/disc. Furthermore, *M. luteus* exhibited reduced susceptibility to these extracts, with the diameter of inhibitory zones measuring 9, 8.5, and 14.25 mm, respectively.

## 3. Materials and Methods

### 3.1. Standards and Reagents

All standards and chemicals were purchased from Sigma Chemical Co. (St. Louis, MO, USA).

### 3.2. Plant Material

The different parts of *P. atlantica* Desf. subsp. *atlantica* (i.e., ripe and unripe fruits, buds, and roots) were collected from tree stations (i.e., Oudjlida, Safsaf, and Ain-Fezza) of Tlemcen province in Algeria, between the end of May and the beginning of June 2016 (ripe fruits were picked up by hand at Oudjlida station in August 2016). To gather the samples, two feet—one male and one female—were considered at each site. Characteristics and details for each sample are listed in Table 8. Samples were air-dried in shadow at room temperature, reduced to fine powder, and stored in the dark until use.

### 3.3. Preparation of Aqueous Extract

The powder (1 g) of each dried sample was resuspended in 30 mL of distilled water for 24 h at room temperature. The extracts were purified by passing through filter papers and evaporated under a vacuum at 50 °C by a Buchi Rotavapor R-200. The dry extracts were weighed and then recovered with distilled water; finally, they were stored at −20 °C until further analysis.

### 3.4. Fruit Oil Extraction

Thirty grams of dry fruit powder were placed in a Soxhlet apparatus, in order to extract the crude plant oil using *n*-hexane (300 mL) as a solvent for 6 h. At the end of extraction, the recovered sample was evaporated using a rotary evaporator at 40 °C. The obtained lipophilic extract was weighed in order to determine the content of oil with respect to the dry weight of the initial matter [44] and stored at +4 °C until the analysis.

### 3.5. Extraction of Phenolic Compounds from P. atlantica Oil

A liquid-liquid extraction technique was used to fraction phenolic components from *P. atlantica* oil, as reported by Pirisi et al. [45]. The researchers dissolved 2 g of oil residue in 1 mL of *n*-hexane and combined it with 2 mL of methanol/water (30:20; *v*:*v*) in a glass tube, and vortexed it for 2 min. After centrifugation at 3000 rpm for 10 min, the methanol layer was collected and the extraction was repeated 2 times on the organic portion. The hydroalcoholic extracts were blended together, washed 2 times with *n*-hexane (2 mL each), evaporated, and resuspended in 2 mL of methanol.

### 3.6. Quantitation of Total Phenolic Content

The Folin-Ciocalteu method was used to determine the total phenolic content [46]. The researchers combined 200 µL of diluted samples with 0.8 mL of 7.5% sodium carbonate and 1.0 mL of a 10-fold diluted Folin-Ciocalteu reagent in test tubes with screw caps. After being vortexed, the tubes were left in the dark for thirty minutes. Using a Specord 200 spectrophotometer, absorption was measured at 765 nm. A calibration curve was created with gallic acid as the standard. The amount of total phenolics in plant dry matter (DM) was expressed as milligrams of gallic acid equivalents (GAE) per gram. The analysis was repeated three times for the reliability of the results.

### 3.7. Total Flavonoid Content

The technique used by Zhishen et al. [47] was used to determine the flavonoid content. A volume of 500 µL of methanol solution at different concentrations of catechin (used as standard for the creation of a quantitation curve), or plant extract, was mixed with 1500 µL distilled water. At time zero, 150 µL of 5% NaNO_2_ was added to the mixture. After 5 min, 150 µL of 10% AlCl_3_ was also joined to the mixture. After incubation for 6 min at room temperature, 500 µL of 1 M NaOH was added. Immediately, the mixture was thoroughly shaken and the absorbance determined at 510 nm against the blank. The total flavonoid content was expressed as mg of catechin equivalents per gram of dry plant matter (mg CE/g DM). Each test was repeated in triplicate.

### 3.8. Content of Condensed Tannins

These metabolites were quantified as proanthocyanidin subunits using the vanillin assay described by Julkunen-Titto [48]. Briefly, 1500 µL of vanillin/methanol solution (4%, *w*/*v*) was added to 50 µL of each extract. Then, 750 µL of concentrated HCl was added and samples were incubated at room temperature for 20 min. The absorbance was measured at 550 nm against a blank. The amount of total condensed tannins was expressed as mg of catechin equivalents per gram of dry plant matter (mg CE/g DM), based on a calibration curve adequately created with an increasing amount of catechin. Three trials per sample were performed.

### 3.9. HPLC-DAD Analysis

Five milliliters of methanol:water (50:50, *v*/*v*) was used for the resuspension of two grams of plant material. Following a ten-fold dilution of the extract in methanol:water (50:50, *v*/*v*), ten microliters were examined. The biochemical profile of *P. atlantica* extract was carried out according to Gismondi et al. [49] method by an HPLC system equipped with an LC-20AD pump, a CBM-20A controller, a SIL-20a HT auto-sampler, and a diode array SPD –M20A (Shimadzu, Japan). In detail, molecules were separated using a Phenomenex Luna 3u C18 (2) column (150 mm × 4.60 mm, 3 µm particle size, Phenomenex-Italy), using a mobile phase consisting of 1% formic acid (*v*/*v*) (phase A) and pure methanol (phase B), at a flow rate of 0.95 mL min^−1^. The elution gradient was set as follows: t 0 min (A 85%, B 15%); t 20 min (A 65%, B 35%); t 55 min (A 10%, B 90%); t 68 min (A 85%, B 15%); t 70 min (end run). Data acquisition was performed using LAB-SOLUTION software (Shimadzu, Kyoto, Japan). Specific secondary metabolites were identified and quantified by comparing their retention time (min), absorption spectrum, and peak area with those of respective pure standards (Sigma-Aldrich, Milan, Italy). Results were expressed as µg of plant metabolite per g of sample dry weight (µg/g D.W.).

### 3.10. GC-MS Analysis

One hundred µL of oil residue extracted from *P. atlantica* ripe fruit were dissolved in 500 µL of hexane. Two microliters were analyzed by a GC-MS instrument (QP2010 Shimadzu, Japan). The chemical separation was performed, in a DB-5 column (30 m × 0.25 mm x 0.25 μm; Agilent Technologies, Santa Clara, CA, USA), setting the conditions of the chromatographic apparatus and mass-spectrometry as widely reported in Nanni et al. [50]. The relative abundance of each molecule was expressed in percentage value with respect to the total mixture (considered as 100%).

### 3.11. Antioxidant Activity

#### 3.11.1. Total Antioxidant Capacity

The phosphomolybdenum technique of Prieto et al. [51] was used to calculate the total antioxidant capacity of the plant extracts. A 0.3 mL aliquot of the sample was combined with 3 mL of the standard reagent, which consisted of 0.6 M sulfuric acid, 28 mM sodium phosphate, and 4 mM ammonium bicarbonate. The reaction mixture was then allowed to cool to room temperature after being incubated at 95 °C for 90 min, and its absorbance at 695 nm was measured. For the analysis of the plant oil, a concentration of 50 μL of sample per mL of hexane/ethanol (*v*/*v*; 4.5:5) was obtained, and then, 150 μL of this mixture was diluted in distilled water to have a final volume of 300 μL. Total antioxidant capacity was expressed as mg of ascorbic acid equivalents per gram of dry matter (mg AAE/g DM). All tests were carried out in triplicate.

#### 3.11.2. DPPH Radical Scavenging Activity Assay

DPPH radical scavenging activity assay was performed as described by Sanchez-Moreno et al. [52]. Fifty microliters of various concentrations of extracts (0.0625–1 mg/mL) were mixed with 1950 µL of a methanolic solution containing 0.025 g/L DPPH (2,2-diphenyl-1-picrylhydrazil). The mixture was shaken vigorously and allowed to stand at room temperature in the dark for 30 min. The absorbance was measured at 515 nm. For plant oil, different concentrations of the sample (i.e., 10, 25, 50, 75, 100, 150, and 200 μL/mL) were prepared in hexane/ethanol (*v*/*v*; 4.5:5). The radical scavenging activity (RSA) was calculated as a percentage of discoloration for the DPPH solution using the following equation:

RSA (%) = (A_blank_ − A_sample_)/A_blank_ × 100

where A_blank_ is the absorbance of the control reaction (containing all reagents except the test compound) and A_sample_ is the absorbance of the solution containing the sample. Each measurement was made in triplicate.

#### 3.11.3. Reducing Power Assay

The reducing power of extracts was calculated using Oyaizu’s method [53]. One milliliter of extracts at different concentrations was combined with 2.5 mL of phosphate buffer (0.2 M, pH = 6.6) and 2.5 mL of potassium ferricyanide water solution (1%; K3[Fe(CN)6]). Following a 20 min incubation period at 50 °C, the mixture was added to 2.5 mL of trichloroacetic acid (10% in aqueous solution). The sample was centrifuged at 3000 rpm for 10 min and the supernatant (2.5 mL), once collected, was added to distilled water (2.5 mL) and to a freshly prepared FeCl_3_ solution (0.5 mL, 0.1%). The absorbance was measured at 700 nm. Ascorbic acid was used as the standard reference. In this test, a high absorbance indicates a high reducing power. The effective concentration EC_50_ (mg/mL) obtained by linear regression analysis and giving an absorbance of 0.5 was used to express the results. All tests were carried out in triplicate.

### 3.12. Antimicrobial Activity

#### 3.12.1. Screening of Antimicrobial Activity

The various plant extracts were tested against six bacteria species, including Gram-positive—such as *Staphylococcus aureus* (ATCC6538), *Listeria monocytogenes* (ATCC19111), and *Bacillus subtilis* (ATCC 6633)—and Gram-negative ones—like *Escherichia coli* (ATCC 8739), *Pseudomonas aeruginosa* (ATCC27853), *Klebsiella pneumoniae* (IBMC Strasbourg); moreover, a yeast was also included, *Candida albicans* (CIP 444). The microbial stocks were revivified and turbidity was adjusted at 0.5 McFarland (D.O = 0.08 to 0.1; λ = 625 nm), which corresponds to 1.2 × 10^8^ UFC/mL for bacteria (O.D = 0.08 to 0.1; λ = 625 nm) and 1.5 × 10^6^ UFC/mL (O.D = 0.12 to 0.15; λ = 530 nm) for yeast [54].

#### 3.12.2. Agar Disc Diffusion Method

The disc diffusion method was used to assess the inhibition zones against the chosen microbial strains, in accordance with the guidelines proposed by the Clinical and Laboratory Standards Institute (CLSI) [55,56]. Bacterial suspensions were prepared in Mueller Hinton Broth (MHB) and yeast suspension in Sabouraud Dextrose Broth (SDB); then, they were incubated at 37 °C for 24 h and at 30 °C for 48 h, respectively. The microorganism suspensions were adjusted to a similar optical density to that of McFarland 0.5 (10^8^ CFU/mL for bacteria and 10^6^ CFU/mL for yeast). Then, each culture was spread on the solid media plates. Whatman no. 1 sterile filter paper disks (6 mm diameter) were impregnated with 400, 1500, and 3000 µg/disc for extract, and crude oil (CO) and its diluted charges (100, 200 µg/disc). Nystatin (100 μg/disc; Sigma) and amphotericin B (100 μg/disc; Sigma) for yeast and ampicillin (10 μg/disc; Biomaxima) and chloramphenicol (30 μg/disc; Sigma Aldrich) for bacteria were served as the positive controls. Inhibition zone diameters (IZDs) (in mm), including the paper disc, were determined after incubation periods of 24 h for bacteria and 48 h for yeast at 37 °C. All tests were carried out in triplicate.

#### 3.12.3. Minimum Inhibitory Concentration (MIC) and Minimum Bactericide (or Fungicidal) Concentration (MBC or MFC)

The microbroth dilution method [57,58] was used to estimate the MIC of extracts in accordance with the Clinical and Laboratory Standards Institute (CLSI) procedures. Every test was conducted in Mueller Hinton Broth. However, *C. albicans* was cultivated on Sabouraud Dextrose Broth (Merck, Darmstadt, Germany). The ultimate density of the microorganism suspensions was adjusted to 10^6^ CFU/mL. Next, a 96-well microplate with 100 µL of extract in each well underwent a 2-fold dilution process to produce a final extract concentration that ranged between 0.019 to 5 mg/mL. The various extract concentrations were dissolved using dimethyl sulfoxide (DMSO), with a final concentration of 1%. MICs were expressed as µg of extract per mL of microorganism culture. MBC/MFC was measured by sub-culturing 10 µL of each well with no visible microbial growth on new culture disks, followed by incubation at 37 °C for 24 h (48 h for the yeast), and identifying the plate with no microbial growth.

### 3.13. Statistical Analysis

All experimental results were performed in triplicate and the data were expressed as means ± standard deviation. The one-way analysis of variance (ANOVA) was used to determine the significance of the findings, and PAST software (4.13) was used to assess the differences using the post hoc lowest standard deviations (LSD) test. Results were considered significant only if associated with *p*-values < 0.05 and, consequently, indicated with different letters.

## 4. Conclusions

Our study investigated how the chemical composition and the antioxidant and antimicrobial properties of the root, bud, and fruit aqueous extracts from *P. atlantica* were influenced by the processed plant organ, gender, and geographical station. Moreover, the plant oil isolated from the fruits of this species was also characterized and studied for the same bioactivities. Our data indicate that *P. atlantica* may be considered a promising source of antioxidants, able to scavenge and neutralize free radicals potentially toxic for human health and pro-inflammatory, and for antimicrobial compounds, more and more essential in fighting drug-resistant infections. Thus, *P. atlantica* tissues could become an optimal natural raw material for extracting novel bioactive metabolites to be used in food, medicine, and cosmetic applications.

## Figures and Tables

**Table 1:** Yields of bud, root, and fruit aqueous extracts from *P. atlantica* harvested in the Oudjlida, Ain-Fezza, and Safsaf regions.

Extract	Yield (%)
Oudjlida male buds	27.162 ± 0.859 ^a^
Oudjlida female buds	35.005 ± 4.631 ^a^
Ain-Fezza male buds	31.485 ± 0.516 ^a^
Oudjlida male roots	8.290 ± 0.975 ^b^
Oudjlida female roots	10.148 ± 0.111 ^b^
Ain-Fezza male roots	6.625 ± 0.728 ^b^
Ain-Fezza female roots	6.96 ± 0.056 ^b^
Safsaf male roots	5.435 ± 0.615 ^b^
Safsaf female roots	8.075 ± 0.318 ^b^
Oudjlida ripe fruits	2.876 ± 0.472 ^c^
Oudjlida unripe fruits	6.88 ± 0.332 ^b^
Safsaf unripe fruits	14.892 ± 1.700 ^d^
Oil from Oudjlida ripe fruits	27.603 ± 1.766 ^a^
Phenolic compounds from oil	0.12 ± 0.014 ^e^

Data represent the mean of three replicates ± SD. The variability among the samples was indicated with different letters only if significant (that is with a *p*-value < 0.05).

**Table 2:** Polyphenolic contents in buds, roots, and fruits aqueous extracts and in the phenolic residue isolated from *P. atlantica* ripe fruit oil.

Extract	Phenolic Content (mg GAE/g DM)	Flavonoid Content (mg CE/g DM)	Condensed Tannin Content (mg CE/g DM)
Oudjlida male buds	284.133 ± 37.640 ^a^	37.677 ± 0.239 ^a^	12.806 ± 0.735 ^a^
Oudjlida female buds	89.254 ± 2.366 ^b^	22.685 ± 0.067 ^b^	14.793 ± 0.821 ^a^
Ain-Fezza male buds	386.785 ± 16.227 ^c^	74.780 ± 9.724 ^c^	12.898 ± 0.301 ^a^
Oudjlida male roots	3.580 ± 1.073 ^d^	1.778 ± 0.213 ^d^	0.294 ± 0.063 ^b^
Oudjlida female roots	18.811 ± 0.214 ^e^	1.093 ± 0.031 ^d^	1.389 ± 0.371 ^b^
Ain-Fezza male roots	16.849 ± 2.992 ^e^	0.709 ± 0.039 ^d^	0.555 ± 0.040 ^b^
Ain-Fezza female roots	13.598 ± 4.903 ^e^	0.561 ± 0.002 ^d^	1.462 ± 0.056 ^b^
Safsaf male roots	12.238 ± 0.123 ^e^	0.652 ± 0.072 ^d^	0.712 ± 0.045 ^b^
Safsaf female roots	38.731 ± 2.270 ^f^	4.341 ± 0.357 ^e^	2.262 ± 0.052 ^b^
Oudjlida ripe fruits	86.337 ± 1.232 ^b^	14.641 ± 1.428 ^f^	0.153 ± 0.017 ^b^
Oudjlida unripe fruits	56.287 ± 1.442 ^g^	9.535 ± 1.034 ^f^	0.624 ± 0.094 ^b^
Safsaf unripe fruits	59.897 ± 0.553 ^g^	6.710 ± 0.047 ^e^	1.163 ± 0.130 ^b^
Phenolic compounds from oil	0.269 ± 0.032 ^h^	0.014 ± 0.005 ^g^	0.037 ± 0.003 ^c^

Values are the mean of three replicates ± SD; DM: dry matter; GAE: gallic acid equivalent; CE: catechin equivalent. The variability among the samples was indicated with different letters only if significant (that is with a *p*-value < 0.05).

**Table 3:** Quantitative analysis of phenols and flavonoids in the aqueous extracts from *P. atlantica* fruits, roots, and buds by HPLC-DAD. Results are expressed as μg of standard equivalent per g of dry plant material (DM). For each secondary metabolite, all measurement results were significant (*p* < 0.05) with respect to the other data of the series.

Identified Compound	Oudjlida Ripe Fruits	Safsaf Female Roots	Ain-Fezza Male Buds
Gallic acid	112.94	0	0
Chlorogenic acid	0.19	3.06	23.15
Caffeic acid	0	1.73	59.17
*p*-Coumaric acid	0	0.13	14.57
Dimethyl-allyl caffeic acid	0	7.28	0
Hydroxytyrosol	12.01	242.70	3998.44
Ellagic acid	0.69	0	42.37
Rutin	6.37	0	60.37
Quercetin-3-*O*-arabinoside	0	0	6.28
Kaempferol-3-*O*-glucoside	0	0	4.86
Quercetin	0	0	0.29

**Table 4:** Chemical components of *P. atlantica* fruit oil detected by GC-MS. All results were reported in percentage as relative abundance (%). The values correspond to the mean of three independent measurements, and for each compound, the maximum deviation registered was always lower than 5% of the respective molecule peak area.

Detected Analyte	Relative Abundance (%)
1,2-Benzenediol, 3,5-bis(1,1-dimethylethyl)-	0.0188
alpha-Pinene	0.0391
Anisole	0.0002
Thujene	0.0085
D-Limonene	0.1909
Artemisia ketone	0.0106
Butanoic acid	0.0108
Sorbitol	0.0193
Isoborneol	0.2304
Tumerone	0.0227
Caproic acid	0.0030
Germacrene D	0.0414
Dodecanol	0.0220
Azulene, 1,2,3,3a,4,5,6,7-octahydro-1,4-dimethyl-7-(1-methylethenyl)-,[1R-(1-alpha,3a-beta,4-alpha,7-beta)]-	0.0988
Hydroxydehydrostevic acid	0.0067
Tetrahydrolinalool	0.0287
Erucic acid	1.1025
Palmitic acid	55.1800
Linolenic acid	1.5955
Stearic acid	1.2052
9-Tetradecenal	0.4813
Oleic acid	3.6240
Ricinoleic acid	0.3185
Anacardol	9.0603
Squalene	8.1807
alpha-Tocopheryl acetate	0.0258
Octadecanal	0.0459
beta-Ocimene	0.0667
Citronellol	0.2370
alpha-Ylangene	0.0467
Arachidic acid	8.2885
Tetradecanol	9.1952
Vitamin E	0.5943

**Table 5:** Antioxidant properties of *P. atlantica* bud, root and fruit aqueous extracts, fruit oil, and phenolic residue from fruit oil.

Extracts	TAC (mg AAE/g DM)	EC_50_ (mg/mL)	
		DPPH	RP
Oudjlida male buds	41.575 ± 0.595 ^a^	0.225 ± 0.007 ^a^	0.094 ± 0.020 ^a^
Oudjlida female buds	36.094 ± 0.957 ^a^	0.305 ± 0.015 ^a^	0.173 ± 0.064 ^b^
Ain-Fezza male buds	51.648 ± 1.659 ^b^	0.129 ± 0.001 ^a^	0.029 ± 0.001 ^c^
Oudjlida male roots	8.221 ± 0.830 ^c^	2.908 ± 0.066 ^b^	0.854 ± 0.022 ^d^
Oudjlida female roots	17.589 ± 0.1077 ^d^	1.740 ± 0.021 ^c^	0.619 ± 0.018 ^d^
Ain-Fezza male roots	6.633 ± 0.193 ^c^	1.713 ± 0.027 ^c^	0.978 ± 0.245 ^d^
Ain-Fezza female roots	6.347 ± 0.375 ^c^	2.778 ± 0.110 ^b^	0.992 ± 0.053 ^d^
Safsaf male roots	8.345 ± 0.146 ^c^	5.206 ± 0.021 ^d^	2.543 ± 0.228 ^e^
Safsaf female roots	18.323 ± 0.358 ^d^	0.106 ± 0.001 ^a^	0.197 ± 0.036 ^b^
Oudjlida ripe fruits	29.070 ± 0.665 ^e^	0.073 ± 0.002 ^a^	0.056 ± 0.002 ^a c^
Oudjlida unripe fruits	17.522 ± 0.328 ^d^	0.332 ± 0.001 ^a^	0.287 ± 0.085 ^b^
Safsaf unripe fruits	13.895 ± 0.538 ^c,d^	0.431 ± 0.016 ^a^	0.132 ± 0.010 ^a^
Oil from Oudjlida ripe fruits	1.67 ± 0.408 ^f^	193.594 ± 28.942 ^e^	103.086 ± 20.540 ^f^
Phenolic residue from oil	0.053 ± 0.008 ^g^	0.524 ± 0.020 ^a^	ND
Ascorbic acid		0.090 ± 0.002	0.063 ± 0.02

Values were the mean of three replicates ± SD; DM: dry matter; RP: reducing power; TAC: total antioxidant capacity; DPPH: 2,2-diphenyl-1-picrylhydrazil; ND: not determined. The variability among the samples was indicated with different letters only if significant (that is with a *p*-value < 0.05).

**Table 6:** Average diameters of the growth inhibition zones (mm) for the different microorganisms cultivated with extracts of *P. atlantica*. Significance (always *p* > 0.05), indicated with different letters, was evaluated among the different samples of the extract on the same conditions (that is per raw).

		Diameter of Inhibition Zone including the Disc (mm ± SD)															
	Organ/ Station	Buds			Roots						Fruits			Oil		Standard	
Strain	Charge (µg/disc)	Oudjlida Male	Oudjlida Female	Ain-Fezza Male	Oudjlida Male	Oudjlida Female	Ain-Fezza Male	Ain-Fezza Female	Safsaf Male	Safsaf Female	Oudjlida Ripe	Oudjlida Unripe	Safsaf Unripe	Charge (µg/disc)	Fatty oil	Amp (10 μg/disc)	Chlor (30 μg/ disc)
Sa	400	0 ± 0	7 ± 0 ^a^	10 ± 0 ^a^	0 ± 0	0 ± 0	0 ± 0	0 ± 0	0 ± 0	0 ± 0	9.5 ± 0.70 ^a^	8.5 ± 0.70 ^a^	0 ± 0	100	0 ± 0	43 ± 1.41 (S)	25.5 ± 0.71 (S)
	1500	9 ± 0 ^a^	15.5 ± 0.70 ^b^	12 ± 0 ^a,b^	12.5 ± 0.70 ^a,b^	12.5 ± 0.70 ^a,b^	10 ± 0 ^a^	10 ± 0 ^a^	14 ± 1.41 ^b^	10 ± 0 ^a^	15 ± 0 ^b^	20.5 ± 0.70 ^c^	10.75 ± 0.35 ^a^	200	0 ± 0		
	3000	9.5 ± 0.70 ^a^	23 ± 0 ^b^	14.5 ± 0.70 ^c^	14.5 ± 0.70 ^c^	13.5 ± 0.70 ^c^	11.5 ± 0.70 ^a c^	14.5 ± 0.70 ^c^	16 ± 1.41 ^c d^	13 ± 0 ^c^	18 ± 1.41 ^d^	24 ± 1.41 ^b^	26 ± 1.41 ^b^	CO	8.5 ± 0.70		
Bs	400	0 ± 0	0 ± 0	8 ± 0 ^a^	0 ± 0	0 ± 0	0 ± 0	0 ± 0	0 ± 0	0 ± 0	0 ± 0	0 ± 0	8 ± 0 ^a^	100	0 ± 0	14 ± 0 (I)	21.5 ± 0.71 (I)
	1500	9 ± 0 ^a^	9 ± 0 ^a^	9.5 ± 0.70 ^a^	12.5 ± 0.70 ^b^	9 ± 0 ^a^	0 ± 0	0 ± 0	0 ± 0	0 ± 0	0 ± 0	0 ± 0	9.25 ± 0.35 ^a^	200	9.5 ± 0		
	3000	9.5 ± 0.70 ^a^	10 ± 0 ^a^	10 ± 0 ^a^	13.5 ± 0.50 ^b^	10.5 ± 0.70 ^a^	0 ± 0	0 ± 0	7.25 ± 0.35 ^a^	0 ± 0	9.5 ± 0.70 ^a^	0 ± 0	10.25 ± 0.35 ^a^	CO	10 ± 0		
Bc	400	0 ± 0	7.5 ± 0.70	0 ± 0	0 ± 0	0 ± 0	0 ± 0	0 ± 0	0 ± 0	0 ± 0	0 ± 0	0 ± 0	0 ± 0	100	0 ± 0	12.25 ± 0.35 (R)	21.5 ± 2.12 (I)
	1500	8.5 ± 0.70 ^a^	8.5 ± 0.70 ^a^	10.5 ± 0.70 ^a^	11.5 ± 0.70 ^a^	11.5 ± 0.70 ^a^	0 ± 0	0 ± 0	0 ± 0	0 ± 0	0 ± 0	0 ± 0	0 ± 0	200	0 ± 0		
	3000	10.5 ± 0.70 ^a^	9 ± 0 ^a^	11 ± 0 ^a^	12.5 ± 0.70 ^a^	12.5 ± 0.50 ^a^	0 ± 0	0 ± 0	0 ± 0	0 ± 0	0 ± 0	0 ± 0	7 ± 0	CO	10 ± 0		
Ml	400	7 ± 0 ^a^	0 ± 0	10.5 ± 0.70 ^b^	0 ± 0	0 ± 0	0 ± 0	0 ± 0	0 ± 0	0 ± 0	0 ± 0	0 ± 0	11 ± 0 ^b^	100	0 ± 0	28 ± 0 (***S***)	46.5 ± 0.71 (***S***)
	1500	9 ± 0 ^a^	9.5 ± 0.70 ^a^	11.5 ± 0.70 ^a^	9.5 ± 0.70 ^a^	0 ± 0	0 ± 0	0 ± 0	0 ± 0	8.75 ± 1.06 ^a^	0 ± 0	0 ± 0	11 ± 1.41 ^a^	200	0 ± 0		
	3000	11 ± 0 ^a^	11.5 ± 070 ^a^	16.5 ± 0.70 ^b^	11.5 ± 0.70 ^a^	0 ± 0	0 ± 0	0 ± 0	0 ± 0	9.5 ± 0.70 ^a^	9 ± 0	8.5 ± 0.70 ^a^	14.25 ± 0.35 ^b^	CO	0 ± 0		
Pa	400	0 ± 0	0 ± 0	0 ± 0	0 ± 0	0 ± 0	0 ± 0	0 ± 0	0 ± 0	0 ± 0	0 ± 0	0 ± 0	0 ± 0	100	0 ± 0	00 ± 0 (R)	00 ± 0 (R)
	1500	10.5 ± 0.70 ^a^	8.5 ± 0.70 ^a,b^	10 ± 0 ^a,b^	0 ± 0	0 ± 0	0 ± 0	0 ± 0	0 ± 0	0 ± 0	7.5 ± 0.70 ^b^	0 ± 0	7 ± 0 ^b^	200	0 ± 0		
	3000	14.5 ± 0.70 ^a^	9.5 ± 0.70 ^b^	11.5 ± 0.70 ^a,b^	0 ± 0	0 ± 0	0 ± 0	0 ± 0	0 ± 0	0 ± 0	12 ± 0 ^a,b^	0 ± 0	9.75 ± 1.06 ^b^	CO	0 ± 0		
Ec	400	0 ± 0	7 ± 0 ^a^	7 ± 0 ^a^	0 ± 0	0 ± 0	0 ± 0	0 ± 0	0 ± 0	0 ± 0	0 ± 0	0 ± 0	0 ± 0	100	0 ± 0	15 ± 1.41 (I)	30 ± 0 (S)
	1500	11.5 ± 0.70 ^a^	8 ± 0 ^a^	9.5 ± 0.70 ^a^	0 ± 0	0 ± 0	0 ± 0	0 ± 0	0 ± 0	0 ± 0	0 ± 0	0 ± 0	6.5 ± 0.5 ^b^	200	0 ± 0		
	3000	14.5 ± 0.70 ^a^	8.5 ± 0.70 ^b^	13.5 ± 0.70 ^b^	0 ± 0	0 ± 0	7.5 ± 0.70 ^b^	11.5 ± 0.70 ^a,b^	9 ± 0 ^b^	0 ± 0	0 ± 0	0 ± 0	7 ± 0 ^b^	CO	0 ± 0		
Kp	400	0 ± 0	0 ± 0	0 ± 0	0 ± 0	0 ± 0	0 ± 0	0 ± 0	0 ± 0	0 ± 0	0 ± 0	0 ± 0	0 ± 0	100	0 ± 0	7.5 ± 0.71 (R)	23 ± 1.41 (S)
	1500	0 ± 0	0 ± 0	0 ± 0	0 ± 0	0 ± 0	0 ± 0	0 ± 0	0 ± 0	0 ± 0	0 ± 0	0 ± 0	0 ± 0	200	0 ± 0		
	3000	0 ± 0	0 ± 0	0 ± 0	0 ± 0	0 ± 0	9.5 ± 0.70 ^a^	0 ± 0	10 ± 0	0 ± 0	0 ± 0	0 ± 0	11.5 ± 0.70 ^a^	CO	0 ± 0		
Ca ATCC	400	0 ± 0	0 ± 0	0 ± 0	0 ± 0	0 ± 0	0 ± 0	0 ± 0	0 ± 0	0 ± 0	0 ± 0	0 ± 0	0 ± 0	100	0 ± 0	38.5 ± 2.12 * (S)	27.5 ± 3.54 ** (S)
	1500	7.5 ± 0.70 ^a^	7 ± 0 ^a^	9.5 ± 0.70 ^a^	0 ± 0	0 ± 0	0 ± 0	0 ± 0	0 ± 0	0 ± 0	0 ± 0	0 ± 0	0 ± 0	200	0 ± 0		
	3000	8 ± 1.41 ^a^	10 ± 1.41 ^a^	11 ± 0 ^a^	0 ± 0	0 ± 0	8.5 ± 0.70 ^a^	0 ± 0	0 ± 0	0 ± 0	0 ± 0	0 ± 0	8 ± 0 ^a^	CO	0 ± 0		
Ca CIP	400	0 ± 0	0 ± 0	0 ± 0	0 ± 0	0 ± 0	0 ± 0	0 ± 0	0 ± 0	0 ± 0	0 ± 0	0 ± 0	0 ± 0	100	0 ± 0	34.5 ± 0.71 * (S)	26 ± 0 ** (S)
	1500	0 ± 0	7.5 ± 0.70 ^a^	14 ± 0 ^b^	0 ± 0	0 ± 0	0 ± 0	0 ± 0	0 ± 0	0 ± 0	7.5 ± 0.70 ^a^	0 ± 0	7.5 ± 0.70 ^a^	200	0 ± 0		
	3000	9 ± 0 ^a^	10.5 ± 0.70 ^a^	15.75 ± 1.06 ^b^	0 ± 0	0 ± 0	0 ± 0	0 ± 0	0 ± 0	0 ± 0	8.5 ± 0.70 ^a^	0 ± 0	7.75 ± 1.06 ^a^	CO	0 ± 0		

* Nystatin (100 μg/disc); ** Amphotericin B (100 μg/disc); CO: Crude Oil; S: Microorganism classified as Susceptible by CLSI criteria to the antimicrobial compound; I: Intermediate; R: Resistant.

**Table 7:** MIC, MBC, and MFC concentrations of *P. atlantica* fruit oil and bud, root, and fruit aqueous extracts. Significance (always *p* > 0.05), indicated with different letters, was evaluated among the different samples of the extract on the same conditions (that is per raw).

		Organ/Station												
		Buds			Roots							Fruits		
Strain	CMI, MBC/MFC(µg/mL)	Oudjlida Male	Oudjlida Female	Ain-Fezza Male	Oudjlida Male	Oudjlida Female	Ain-Fezza Male	Ain-Fezza Female	Safsaf Male	Safsaf Female	Oudjlida Ripe	Oudjlida Unripe	Safsaf Unripe	Fatty Oil From
*S. aureus*	CMI	1172 ^a^	146.50 ^b^	36.62 ^c^	146.50 ^b^	>75,000 ^d^	18,750 ^f^	293 ^g^	2344 ^h^	37,500 ^i^	293 ^g^	73.25 ^l^	37,500 ^i^	122.05 ^b^
	CMB	>75,000 ^a^	146.50 ^b^	293 ^c^	>75,000 ^a^	>75,000 ^a^	75,000 ^a^	75,000 ^a^	>75,000 ^a^	>75,000 ^a^	>75,000 ^a^	>75,000 ^a^	>75,000 ^a^	>75,000 ^a^
*B. subtilis*	CMI	1172 ^a^	1172 ^a^	36.62 ^b^	293 ^c^	>75,000 ^d^	/	/	9375 ^e^	/	4688 ^f^	/	18,550 ^g^	62,500 ^h^
	CMB	>75,000 ^a^	75,000 ^a^	293 ^b^	>75,000 ^a^	>75,000 ^a^	/	/	>75,000 ^a^	/	>75,000 ^a^	/	>75,000 ^a^	>75,000 ^a^
*B. cereus*	CMI	586 ^a^	586 ^a^	36.62 ^b^	589 ^a^	75,000 ^c^	/	/	/	/	/	/	18,550 ^d^	15,625 ^e^
	CMB	75,000 ^a^	>75,000 ^a^	75,000 ^a^	>75,000 ^a^	>75,000 ^a^	/	/	/	/	/	/	>75,000 ^a^	125,000 ^a^
*M. luteus*	CMI	146.50 ^a^	3663 ^b^	36.62 ^c^	/	/	/	/	/	2344 ^b^	9375 ^d^	2344 ^b^	9375 ^d^	/
	CMB	4688 ^a^	75,000 ^b^	37,500 ^c^	/	/	/	/	/	>75,000 ^b^	>75,000 ^b^	>75,000 ^b^	75,000 ^b^	/
*P. aeruginosa*	CMI	1172 ^a^	1172 ^a^	293 ^b^	/	/	/	/	/	/	293 ^b^	/	37,500 ^c^	/
	CMB	>75,000 ^a^	75,000 ^a^	1172 ^b^	/	/	/	/	/	/	>75,000 ^a^	/	>75,000 ^a^	/
*E. coli*	CMI	2344 ^a^	2344 ^a^	4688 ^b^	/	/	293 ^c^	146.50 ^d^	2344 ^a^	/	/	/	2344 ^a^	/
	CMB	>75,000 ^a^	18,750 ^b^	>75,000 ^a^	/	/	>75,000 ^a^	>75,000 ^a^	>75,000 ^a^	/	/	/	>75,000 ^a^	/
*K. pneumoniae*	CMI	/	/	/	/	/	2344 ^b^	/	18,750 ^b^	/	/	/	37,500 ^c^	/
	CMB	/	/	/	/	/	>75,000 ^a^	/	>75,000 ^a^	/	/	/	>75,000 ^a^	/
*C. albicans* *ATCC* 10231	CMI	3663 ^a^	3663 ^a^	75,000 ^b^	/	/	/	/	/	/	/	/	4688 ^a^	/
	MFC	>75,000 ^a^	75,000 ^a^	>75,000 ^a^	/	/	/	/	/	/	/	/	>75,000 ^a^	/
*C. albicans* CIP 444	CMI	3663 ^a^	3663 ^a^	1172 ^b^	/	/	4688 ^a^	/	/	/	2344 ^c^	/	2344 ^c^	/
	MFC	>75,000 ^a^	>75,000 ^a^	18,750 ^b^	/	/	>75,000 ^a^	/	/	/	>75,000 ^a^	/	>75,000 ^a^	/

**Table 8:** Details of the studied plant materials.

Stations	Site of Harvest	Plant Parts	Month of Harvest
Oudjlida	Longitude: 1°20′10.8′′ W Latitude: 34°55′06.6′′ N Altitude: 500 m	Male Buds Female Buds Male Roots Female Roots Ripe fruits Unripe fruits	End of May 2016
			End of May 2016
			End of May 2016
			End of May 2016
			August 2016 End of May 2016
Safsaf	Longitude: 1°16′ 33′′ W Latitude: 34°53′ 59′′ N Altitude: 616 m	Male Roots Female Roots Unripe fruits	End of May 2016 End of May 2016 End of May 2016
Ain-Fezza	Longitude: 1°14′18” W Latitude: 34°52′45” N Altitude: 730 m	Male Roots Female Roots Male Buds	Beginning of June 2016
			Beginning of June 2016
			Beginning of June 2016

## Data Availability

All data generated or analyzed during this study are included in this published article.
